# Integrating Resonator to Enhance Magnetometer Microelectromechanical System Implementation with ASIC Compatible CMOS 0.18 μm Process

**DOI:** 10.3390/mi12060635

**Published:** 2021-05-29

**Authors:** Chih-Hsuan Lin, Chao-Hung Song, Kuei-Ann Wen

**Affiliations:** Department of Electronic Engineering, National Chiao Tung University, Hsinchu 300, Taiwan; stellawen@mail.nctu.edu.tw (C.-H.S.); nonox1257@gmail.com (K.-A.W.)

**Keywords:** microelectromechanical systems, three-axis magnetometer, three-axis accelerometer

## Abstract

In this study, a multi-function microelectromechanical system (MEMS) was integrated with a MEMS oscillator, using the resonant frequency oscillation characteristics of the oscillator to provide the Lorentz current of the magnetometer to enhance a large dynamic range of reading, which eliminates the off-chip clock and current generator. The resonant frequency can be adjusted by adjusting the bias voltage of the oscillator to further adjust the sensitivity of the magnetometer. With the mechanical *Q* value characteristic, a great dynamic range can be achieved. In addition, using the readout circuit of the nested chopper and correlated double-sampling (CDS) to reduce the noise and achieve a smaller resolution, the calibration circuit compensates for errors caused by the manufacturing process. The frequency of the tuning range of the proposed structure is 17,720–19,924 Hz, and the tuning range of the measurement result is 110,620.36 ppm. The sensitivities of the x-, y-, and z-axes of the magnetometer with driving current of 2 mA are 218.3, 74.33, and 7.5 μV/μT for ambient pressure of 760 torr. The resolutions of the x-, y-, and z-axes of the magnetometer with driving current of 2 mA are 3.302, 9.69, and 96 nT/√Hz for ambient pressure of 760 torr.

## 1. Introduction

In recent years, the magnetometer (MAG) has become one of the key elements in the inertial measurement unit (IMU), used for navigation, alignment, height detection, and so on. The signal of angular velocity can be obtained by the MAG sensor with the use of software [1]. With this, the conventional 9-axis IMU can be implemented without requiring a gyroscope. In the natural environment, received signals come from different magnitudes and frequency ranges. Therefore, how to design a wide range of MAGs becomes more important for wearable device applications.

This paper refers to the previous paper titled “A. Multi-function microelectromechanical Systems Implementation with ASIC compatible CMOS 0.18 μm process” [2]. In the previous paper, the microelectromechanical systems (MEMS) with multi-functions including a three-axis MAG, a three-axis accelerometer (ACC).

Then, the magnetic sensor has many advantages to replace the MEMS gyroscope and reduces hardware cost. However, the resolution was 44.06–87.46 nT/√Hz and was limited by circuit noise equivalent magnetic field (CNEM) and the equivalent Brownian noise (BNEA). Moreover, the curvature was still too large and the sensitivity was too small in the three-axis MAG, at 7.1–10.7 uV/uT.

In this paper, the length of the beam is decreased in the MEMS to reduce the curvature and sacrifice sensitivity. To solve the problems of the sensitivity falling and the problems encountered in the previous paper, the MAG-integrated resonator is used.

The MAG with Lorentz current integrates a microelectromechanical system (MEMS) resonator to replace the original DC bias current. Thus, the MAG and the MEMS resonator resonate at close resonance frequency to expand the sensitivity tuning range.

In the proposed MEMS oscillator with fishbone structure, the resonance frequency has a large tuning range and can provide Lorentz current with different frequencies. It can adjust the sensing range of the MAG from microtesla to tesla values and is suitable for many applications. The MEMS oscillator with fishbone is analyzed by changing the DC between sense and shuttle to enhance the softening effect of the spring [3]. Moreover, the entire circuit can be simply divided into three parts. The first part is a readout circuit including noise-reducing chopper architecture, frequency division multiplexing, and time division multiplexing methods. Then the calibration circuit eliminates the output DC offset due to process changes. Finally, the Lorentz current generator is composed of a MEMS oscillator and a sustaining amplifier.

The design specifications indicate a sensing range of the MAG for ambient pressure of 760 torr.

The development of a multi-axis complementary metal-oxide semiconductor (CMOS) MEMS resonant magnetic sensor using Lorentz and electromagnetic forces was presented in [4] and used in-plane coils to drive a suspended spring-mass structure and produced input AC current. An ultra-sensitive Lorentz force MEMS MAG with a pico-tesla limit of detection was presented in [5] and the input bias current was 7.245 mA. Integrated fluxgate MAG for use in isolated current sensing was presented in [6] and used a Forster-type fluxgate with simplified excitation current. A z-axis MAG for MEMS inertial measurement units using an industrial process was presented in [7] and its overall sensitivity of 150 μV/μT at 250 μA of peak driving current was still small. A real-time 32.768 kHz clock oscillator using a 0.0154 mm^2^ micromechanical resonator frequency-setting element was presented in [8] and reduced power consumption, operating with only 2.1 μW of DC power. A sub-150 microwatt back-end-of-line (BEOL)-embedded CMOS-MEMS oscillator with a 138 dBΩ ultra-low-noise trans-impedance amplifier (TIA) was presented in [9] and the phase noise figure-of-merit of 190 dB was achieved at 1-kHz offset with a resonator *Q* of 1900. Phase-noise reduction in a CMOS-MEMS oscillator under nonlinear MEMS operation was presented in [10] and analyzed a phase-controlled closed-loop and the frequency stability of the self-sustained oscillator.

## 2. Materials and Methods

### 2.1. Process, Modeling, and Design of MEM Sensor, and Structure of MEMS Magnetometer

The proposed multi-function MEMS, including magnetometer (MAG) and accelerometer (ACC) functions, was fabricated with the standard UMC 0.18 μm 1-poly, 6-metal (1P6M) CMOS-MEMS process. The typical differential equation of mechanical equilibrium is given in [11]. The frequency response of displacement to external actuation force is $\frac{x\left(s\right)}{F_{ext}\left(s\right)}=\frac{k_{e}{}^{-1}}{\left(1-\frac{w^{2}}{w_{0}^{2}}\right)+\frac{jw}{Qw_{0}}}$, quality factor *Q* is *Q* = $\frac{\sqrt{m_{e\;}k_{e}}}{b_{e}}$, natural frequency *ω*_0_ is $w_{0}=\sqrt{\frac{k_{e}}{m_{e\;}}}$, and Hooke’s law is given as $F_{ext}=k_{e}x\;if\;w<<w_{0}$, where *m_e_* is the effective mass and x is its displacement, *k_e_* is an effective spring constant, *b_e_* is an effective damping coefficient, and $F_{ext}$ is any external actuation force.

When an actuation force operates at the natural frequency $\omega_{0}$, the magnitude response of displacement becomes $\left|x\left(j\omega_{0}\right)\right|=\frac{Q\left|F_{ext}\left(j\omega_{0}\right)\right|}{k_{e}}$. This amplification of the displacement magnitude response by *Q* times at resonance frequency is one of the most typical characteristics of the resonator and is used to expand the sensitivity tuning range of the MAG in this paper.

**Figure 1:**
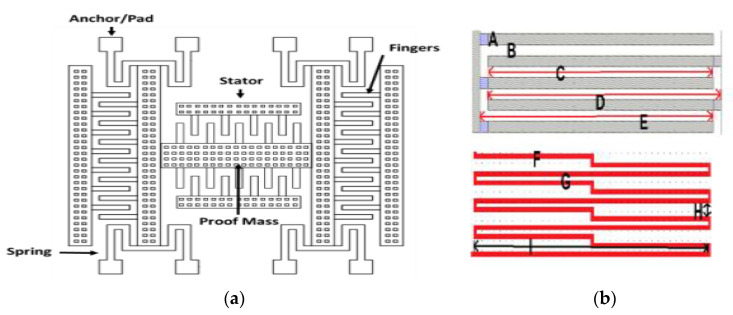
(**a**) Model of microelectromechanical (MEMS) magnetometer; (**b**) marks on combs and springs of the structure.

The model of the MEMS MAG includes stator, rotor, anchors, fingers, springs, and proof mass, and is shown in Figure 1a. The length and number of folding springs [12] will influence the structure of the resonance frequency. In addition, the length of the beam is decreased in the structure to reduce the curvature. The sizes of the structure’s different parts are shown in Table 1 and are marked in Figure 1b, and the resolution of the UMC 0.18 μm CMOS MEMS process is 0.001 μm. In comparison to the previous paper [2], the sizes of the structure are reduced to improve the curvature of the structure.

### 2.2. Operational Principle of MEMS Magnetometer

**Figure 2:**
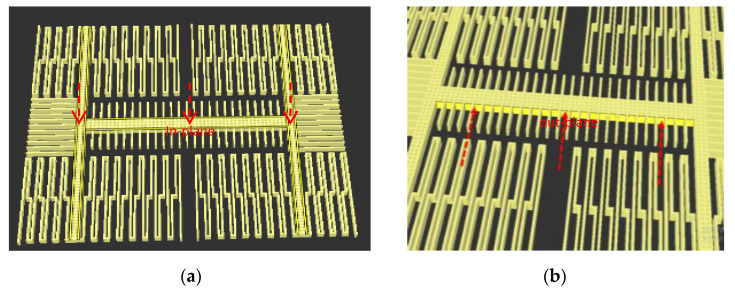
(**a**) Model of sensing x- and y-axis (in-plane) magnetic field; (**b**) model of sensing z-axis (out-plane) magnetic field.

The magnetic field $\vec{B}\left(j\omega \right)$ with applied current $\vec{I}\left(j\omega \right)$ flowing through a suspended conductor of length L will result in the Lorentz force $\vec{F_{mag}}\left(j\omega \right)$, which can be shown as $\vec{F_{mag}}\left(j\omega \right)=LI\left(\vec{l}\left(j\omega \right)\times \vec{B}\left(j\omega \right)\right)$ [13]. The suspended conductor of length L is designed to be 642.2 μm to increase the length of the current flow and obtain greater sensitivity, and the width of the suspended conductor is 2.6 μm. This structure widens the beam area to reduce the cross-axis interference phenomenon and uses the time-sharing method to reduce mutual interference when the x, y, and z currents are applied. Models of sensing the in-plane and out-plane magnetic fields are shown in Figure 2a,b.

## 3. Simulation Results of Finite Element Method for MEMS Sensor

**Figure 3:**
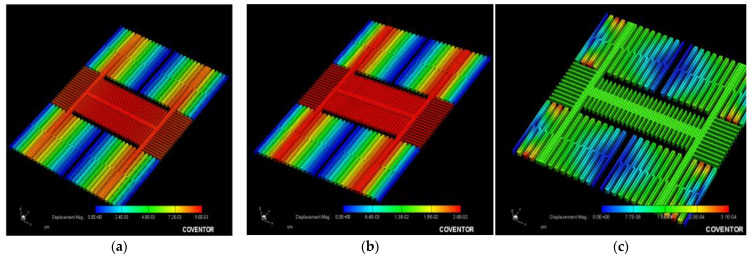
Magnetometer shows plane motion direction for detecting (**a**) y-axis, (**b**) x-axis, and (**c**) z-axis.

A 3-D model of the MAG is established by the CoventorWare tool, and the element unit is $0.5\times 0.5\times 0.5\;\left({\mu m}^{3}\right)$. The resonance frequency $f_{O}$, through modal analysis by MemMech, analyzes displacement and resonance frequency; the x-, y-, z-axis resonance frequencies are 10.059, 17.555, and 18.776 kHz, generalized masses are 9.51, 3.95, and 3.75 $\times 10^{-10}\;\mathrm{kg}$, and the structure at the resonance frequency will have *Q* times higher displacement than the original displacement and greater sensitivity. By the movement displacement of the x-, y-, z-axis modal analysis, we can ensure the design’s current direction and the MAG’s plane motion direction along the x-, y-, and z-axes, as shown in Figure 3.

Since the applied magnetic force is converted to pressure that the CoventorWare tool can simulate, the expected applied current is 2 mA [6,14]. Pressure is applied to the beam with a magnetic field of 0–6000 μT with an interval of 400 μT to simulate deformation for detecting the magnetic field in the in-plane and out-plane direction; the displacement is 0.0153/- μm and 0.0005/0.000167 μm and the sensitivity is approximately $2.55\times 10^{-7}/-\frac{\mu m}{\mu T},\;8.33\times 10^{-8}/2.783\times 10^{-8}\;\frac{\mu m}{\mu T}$.

**Figure 4:**
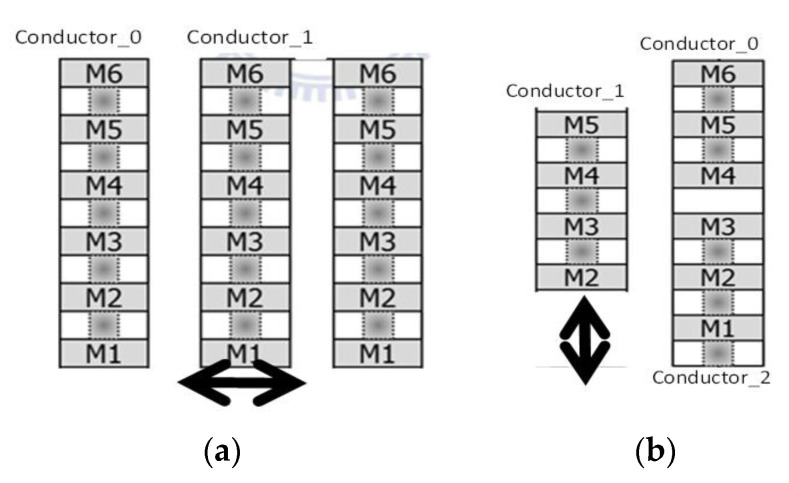
Capacitors for detecting magnetic field in (**a**) z direction and (**b**) x and y directions.

The MAG moves in the x- or y-axis direction; conductor_0 (stator) and conductor_1 (rotor) stacked METAL1–5 are shown in Figure 4a, the initial capacitance is $C_{0}$ = 1.103×50×0.8=44.12 fF, the number of fingers is 40, and the overlap part of the finger is 80% of the original. When detecting the x- or y-axis magnetic field, the MAG moves in the out-plane direction; with conductor_1 (rotor) stacked METAL2–5 and conductor_0 with METAL1–3 and conductor_2 with METAL4–5 as the stator, the average initial capacitance value is $C_{0}=\left[\frac{1.001+1.229}{2}\right]\times 0.8\times N=14.41$ fF (Figure 4b), and N = 18, which corresponds to the x- and y-axes.

Displacement (d) and capacitance variance (ΔC) are equal to $C_{0}\times \frac{\Delta d}{d}$. When detecting a z-axis magnetic field of 6000 μT, the displacement and capacitance variance of the MAG along the x-axis at the frequency $f_{O}$ are 256 nm and 1.815 fF, and in the y-axis direction are 53.6 nm and 0.624 fF. When detecting an x-axis magnetic field of 6000 μT, the displacement and capacitance variance of the MAG along the z-axis at resonance are 30.8 nm and 0.058 fF. Then, we calculate the three-axis capacitance change values for 16 g acceleration and at resonance frequency:$\;\Delta C_{x}=0.0205\;\mathrm{fF},$ $\Delta C_{y}=0.0205\;\mathrm{fF},$ and $\Delta C_{z}=0.296\;\mathrm{fF}$.

Because the *Q* value has an important influence on the structure, the general damping constant is divided into squeeze and slide [11]. The viscosity of air at 760 torr and 300 K is 1.86 × 10^−5^ kg/ms and is set as the default value in the simulator, according to [15,16].

Then, finite element method (FEM) simulation software can be used to simulate the damping coefficient of the structure’s out-plane motion; the slide damping coefficient under an out-plane motion for ambient pressure of 760 torr is dominantly affected, and is about $6.874\times 10^{-7}\left(\frac{N}{\frac{m}{s}}\right)$ at the resonance frequency. The *Q* value can be estimated as follows: $Q=\frac{2\pi f_{0}M}{b}$, where $f_{0}$ is the resonance frequency, *M* is the mass, and $k=\frac{F}{x}=\frac{0.0024335\left(\mu N\right)}{0.00016742\left(\mu m\right)}\sim 14.535$, $Q_{out-plane}=\frac{\sqrt{mk}}{b}=\frac{1.069\times 10^{-4}}{6.874\times 10^{-7}}\sim 155.57$.

When moving in the in-plane motion along the y-axis, the squeeze and slide coefficients for the in-plane motion for ambient pressure of 760 torr are $b=4.433052\times 10^{-7}+2.548642\times 10^{-6}\sim 2.992*10^{-6}\left(\frac{N}{\frac{m}{s}}\right)$, $k=\frac{F}{x}=\frac{0.0024335\left(\mu N\right)}{0.00016742\left(\mu m\right)}\sim 14.535$ $\left(\frac{N}{m}\right)$, $Q_{in-plane}=\frac{\sqrt{mk}}{b}\sim 25.3$.

Finally, the equivalent Brownian noise for ACC is $1.44615\;\frac{\mu g}{\sqrt{\mathrm{Hz}}}$ [17]. In addition, the CoventorWare tool is used to simulate the applied pressure and convert it into force (*F*) and displacement (*x*), and according to Hooke’s law *F* = *kx*, the x- and z-axis spring constants are calculated, shown by $k_{x}=0.875\;\frac{N}{m}$, $k_{z}=4.0446\;\frac{N}{m}$. The Brownian noise equivalent magnetic field (BNEM) for the x-, y-, and z-axes of the MAG for ambient pressure of 760 torr are shown by ${\mathrm{BNEM}}_{x}=32.78\frac{\mathrm{nT}}{\sqrt{\mathrm{Hz}}}$, ${\mathrm{BNEM}}_{y}=99.93\frac{\mathrm{nT}}{\sqrt{\mathrm{Hz}}}$, and ${\mathrm{BNEM}}_{z}=257.53\frac{\mathrm{nT}}{\sqrt{\mathrm{Hz}}}$ [18].

## 4. Simulation Results of Finite Element Method for MEMS Resonator

### 4.1. Operational Principle of MEMS Resonator

**Figure 5:**
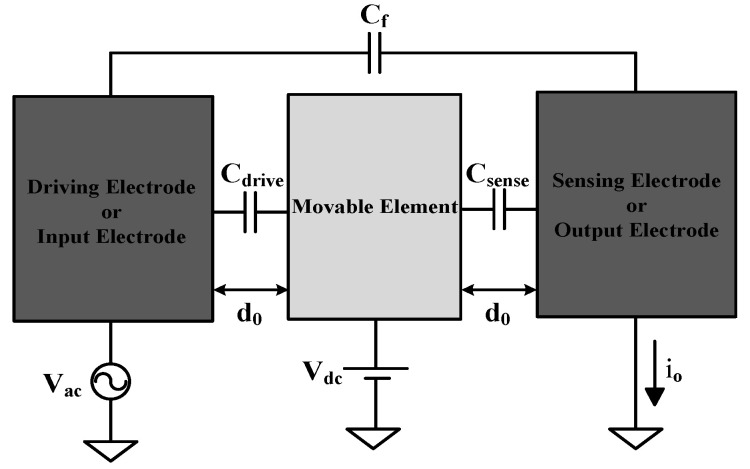
Model of general electrostatic MEMS transducer resonator.

Figure 5 shows a fully differential electrostatic MEMS transducer resonator composed of driving electrodes, movable elements, and sensing electrodes. Assuming that the area of cross-section A is the overlap between rotor and stator, gap *d*_0_ is the spacing when the MEMS is in the neutral position and ε is the permittivity of the material. If there is a displacement x between stator and rotor, the capacitance is given by $C_{drive}=\frac{\varepsilon A}{d_{0}\pm x}$ $C_{sense}=\frac{\varepsilon A}{d_{0}\mp x}$, where $C_{dc}$ is the DC bias voltage applied to the movable element. If we let $V_{ac}$ = 0 V, the electrostatic force that attracts the moving part to both driving and sensing electrodes is given by $F_{drive}=F_{sense}=\frac{-\partial \left(\frac{C_{drive}V_{dc}^{2}}{2}\right)}{\partial x}=\frac{-1}{2}\times \frac{\partial C_{drive}}{\partial x}\times V_{dc}^{2}$.

Because the structure is symmetric along the x-axis, the electrostatic forces of the sensing and driving parts will cancel each other out and the structure will be in equilibrium. Then, if we add the non-zero AC voltage to the driving electrode and assume that |*V_dc_*| >> |*V_ac_*|, the force due to the driving electrode will become $F_{drive}=\frac{-\partial \left(\frac{C_{drive}\times {\left(V_{dc}-V_{ac}\right)}^{2}}{2}\right)}{\partial x}≅\frac{-1}{2}\times \frac{\partial C_{drive}}{\partial x}\times V_{dc}^{2}++\frac{\partial C_{drive}}{\partial x}V_{dc}V_{ac}$ and the first part will be cancelled by the electrostatic force caused by the sensing electrode. The total actuating force can be expressed as $F_{actuation}=\frac{\partial C_{drive}}{\partial x}V_{dc}V_{ac}$ and the system will become a resonator and reach the maximum displacement dependent on the frequency and *Q* factor of the MEMS structure.

The output current $i_{o}$ is shown in Figure 4, and the feedthrough current caused by $C_{f}$ and the output current can be determined by $i_{o}=\frac{\partial Q}{\partial t}=\frac{\partial \left(CV\right)}{\partial t}=V_{dc}\frac{\partial C_{sense}}{\partial t}+C_{f}\frac{\partial V_{ac}}{\partial t}$; the former part is motional current caused by the movement of the rotor and the last term is the undesired part originated from feedthrough capacitance. We set the frequency of driving voltage to resonance frequency $f_{o}$ to obtain the largest $\frac{\partial C_{drive}}{\partial x}$ term and maximize the actuating force. Additionally, we generally set the *C_f_* much smaller than $C_{sense}$ to validate the lack of feedthrough current. When the structure is operated under resonance frequency, it can be approximately changed to $i_{o}≅V_{dc}\frac{\partial C_{sense}}{\partial t}=V_{dc}\frac{\partial C_{sense}}{\partial x}\frac{\partial x}{\partial t}=V_{dc}\frac{\partial C_{sense}}{\partial x}f_{0}x$, where *f*_0_ is the resonance frequency of the moving structure and x is the displacement.

The value of $C_{sense}$ is given by $\frac{\partial C_{sense}}{\partial x}≅\frac{\varepsilon_{0}N_{gn}h_{r}\zeta }{d_{0}}$ [19], where *N_gn_* denotes the number of gaps in comb fingers; ζ is equal to 1.1 and is the constant to model the capacitance due to fringing electric fields; $\varepsilon_{0}$ is the permittivity constant; $h_{r}$ is the thickness of the structure, which is the total thickness stacked from METAL1 to METAL6; and $d_{o}$ is the gap spacing between rotor and stator. Thus, by substituting the parameters, the output current can be given as $i_{o}≅\frac{{\left(V_{dc}N_{gn}\varepsilon_{0}h_{r}f_{0}\right)}^{2}V_{ac}Q}{d_{0}{}^{2}k_{e}}$, and the frequency driving voltage *V_ac_* is on its resonance frequency *f*_0_.

### 4.2. Structure of MEMS Resonator

**Figure 6:**
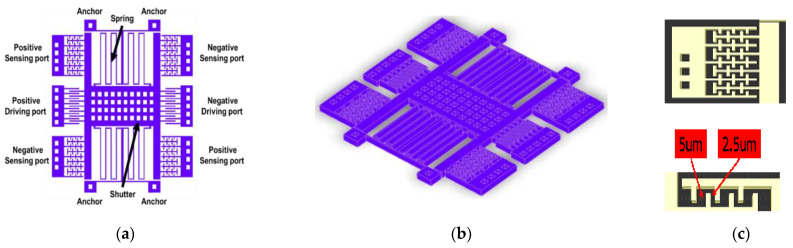
(**a**) Top view with illustration; (**b**) oblique view of MEMS oscillator; (**c**) fingers on each side and distance of gaps between stator and rotor.

A fully differential MEMS oscillator structure was implemented, as shown in Figure 6a. The prototype of this MEMS oscillator is based on [20]. This structure consists of two driving ports, two sensing ports, and a movable shutter. The shutter is suspended above the substrate by four symmetrical springs with two folds, each connected to four anchors through clamp–clamp beams instead of directly connecting the springs to the anchors.

The structure of the shutter and electrodes is stacked from METAL1–6 to increase the variance of capacitance. DC bias *V_dc_* is supplied to the shutter through the surrounding anchor. The geometric parameters of the structure are shown in Table 2.

To minimize the feedthrough capacitance [21], the proposed MEMS oscillator was designed, and different views are shown in Figure 6a,b. The electrodes were changed from a comb to fishbone design. The purpose of the design is to solve the problem of feedthrough current and increase the tuning range of the resonance frequency as the bias voltage is varied.

The structure is symmetric along the oblique diagonal line. Fingers on each side are set with different gap spacings, as shown in Figure 6c. In this way, simulation results will show the sensitivity and the effect of spring softening effect will increase. A zoomed-in view of the fingers is shown in Figure 6c. There are two different gap spacings between rotor and stator, 2.5 and 5 μm, which gives an unbalanced force from the beginning to make it offset on the x-axis. When the AC drive signal is applied, the spring on the offset side is softened and produces a large change in resonance frequency. The driving electrodes have the remaining comb design to maintain the linearity of the driving force. The sizes of the proposed MEMS structure are shown in Table 2.

### 4.3. Model of MEMS Resonator

**Figure 7:**
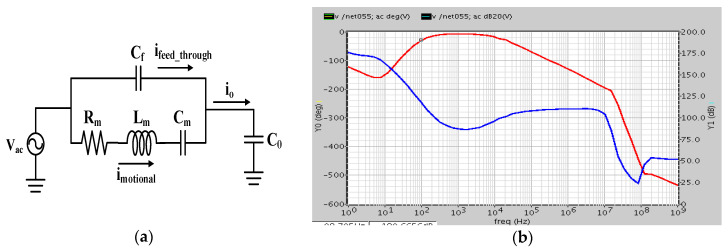
(**a**) Equivalent resistor, inductor, capacitor (RLC) model of MEMS oscillator; (**b**) Bode plot of loop gain and phase with large feedthrough capacitance.

The MEMS oscillator can be modeled as an equivalent series resistor, inductor, and capacitor (RLC) circuit in parallel with a feedthrough capacitor, *C_f_* [19,22], as shown in Figure 7a. To evaluate the effective *R*, *L*, and *C* values, the ratio of electromechanical transformer turns *η_e_* is defined by actuating force *F_act_* over driving voltage *V_ac_*, and $\eta e=\frac{F_{act}}{V_{ac}}=\frac{\varepsilon_{0}N_{\mathrm{gn}}h_{r}\zeta V_{dc}}{d_{0}}$. With an effective transformer turn ratio, the equivalent RLC can be obtained, given by $R_{m}=\frac{b_{e}}{{\eta_{e}}^{2}}=\frac{d_{0}^{2}\sqrt{m_{e}k_{e}}}{Q\zeta^{2}N_{gn}{}^{2}\varepsilon_{0}{}^{2}h_{r}{}^{2}V_{dc}{}^{2}}$, $L_{m}=\frac{m_{e}}{{\eta_{e}}^{2}}=\frac{d_{0}{}^{2}m_{e}}{\zeta^{2}N_{gn}{}^{2}\varepsilon_{0}{}^{2}h_{r}{}^{2}V_{dc}{}^{2}}$,$\;C_{m}=\frac{{\eta_{e}}^{2}}{k_{e}}=\frac{\zeta^{2}N_{gn}{}^{2}\varepsilon_{0}{}^{2}h_{r}{}^{2}V_{ac}{}^{2}}{d_{o}{}^{2}k_{e}}$, where $m_{e}$, $k_{e}$, $b_{e}$, and *Q* are the equivalent mass, spring constant, damping coefficient, and quality factor, respectively. $C_{o}$ is the capacitance between the moving part and sensing electrode as the shutter is in equilibrium.

$R_{m}$ is motional impedance, which indicates the power loss in the MEMS structure with large $R_{m}$, which means the following sustaining amplifier must have high gain. Feedthrough $C_{f}$ gets larger and larger compared to $C_{m}$; the gain and phase plot shown in Figure 7b will be critically influenced and cannot satisfy Barkhausen’s criterion at the resonance frequency and the oscillator may fail to oscillate.

### 4.4. Modeling and FEM Simulation of Proposed MEMS Oscillator

We conducted the modal analysis of the proposed MEMS structure in MemMech, and the resonance frequencies were 16.41, 18.79, and 21.96 kHz, and the generalized masses were 2.14, 6.61 and 3.46 × $10^{-10}\;\mathrm{kg}$. Then, the initial capacitance between stator and rotor and the feedthrough capacitance between driving and sensing ports were simulated in MemElectro, which can set the electrostatic boundary conditions to simulate the charge and capacitance between conductors. A summary of the capacitance array is shown in Table 3, and it can be seen that the feedthrough capacitance from positive and negative driving ports to sensing ports is nearly the same, which means *C_feed_*_1_ is equal to *C_feed_*_2_ and the feedthrough current can theoretically be cancelled.

Finally, the damping coefficient was simulated in DampingMM in CoventorWare, and the squeeze damping and slide damping coefficients were 1.9948 × 10^−7^ and 2.1713 × 10^−7^ N/(m/s) for ambient pressure of 760 torr.

After finishing the FEM simulation, the resonance frequency was 18.796 kHz, effective mass in the desired mode was 6.6108 × 10^−10^ kg, squeeze damping coefficient was 1.9948 × 10^−7^ N/m/s, slide damping coefficient was 2.1713 × 10^−7^ N/m/s, total damping coefficient was 4.1661 × 10^−7^ N/m/s, effective spring constant was 9.22 N/m, and feedthrough capacitance was 1.156 fF.

**Figure 8:**
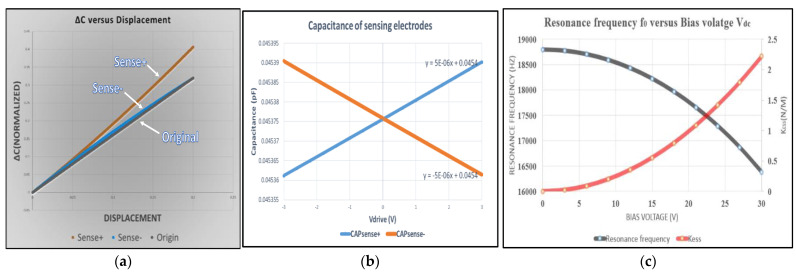
(**a**) Normalized variant capacitance versus displacement of MEMS structure; (**b**) capacitance versus driving voltage of the proposed structure; (**c**) resonance frequency and electrostatic stiffness versus bias voltage.

With mechanical parameters, we can evaluate *η_e_*, $R_{m}$, $C_{m}$, and $L_{m}$. As ζ, $V_{dc}$, $h_{r}$, and $N_{gn}$ were set to 1.1, 20 V, 11.14 μm, and 28, respectively, electromechanical transformer turn ratio *η_e_* will be 2.34 × 10^−8^ C/m. As a result, $R_{m}$, $L_{m}$, and $C_{m}$ are 760 M, 1.2 MH, and 59.2 aF, respectively. The RLC equivalent model and important parameters under 760 torr are shown in Figure 8a; $C_{feed}$ is 1.156 fF, $C_{1}$ is 21.555 fF, $C_{2}$ is 45.374 fF, $R_{m}$ is 760 MΩ, $L_{m}$ is 1.2 MH, and $C_{m}$ is 7600.0593 fF.

In addition, quality factor *Q* is an important specification in the oscillator. Under ambient pressure of 760 torr, the quality factor can be calculated as $\frac{\sqrt{mk}}{b}$, equal to 187.5.

The proposed MEMS structure has larger (∂Csense)/∂x than the MEMS structure without fishbone (original). Neglecting the fringing capacitance, the variant capacitance of sensing electrodes can be expressed as $\Delta C_{sense+}=2\times \left(3\times \frac{\varepsilon A}{5+\Delta x}+3\times \frac{\varepsilon A}{2.5-\Delta x}\right)-2\times \left(3\times \frac{\varepsilon A}{5}+3\times \frac{\varepsilon A}{2.5}\right)$ and $\Delta C_{sense-}=2\times \left(3\times \frac{\varepsilon A}{5-\Delta x}+3\times \frac{\varepsilon A}{2.5+\Delta x}\right)-2\times \left(3\times \frac{\varepsilon A}{5}+3\times \frac{\varepsilon A}{2.5}\right)$, where Δx is the displacement and A is the overlapping area.

As seen in Figure 8, the displacement gets larger and the nonlinearity in the proposed MEMS structure increases. By operating the MEMS structure in the linear region, at the same area, the proposed MEMS has a larger capacitance sensitivity compared to the original.

The variance of capacitance in the proposed structure is 1.25 times larger than the original. The magnification can be even larger by lengthening the fishbone finger.

The second advantage of the proposed structure is the wide tuning range of resonance frequency with a strong spring softening effect. Assuming that $V_{dc}$ and $V_{ac}$ are fixed, (*∂Cdrive*)/*∂x* will determine the magnitude of electrostatic spring constant $K_{ess}$. The resonance frequency, which is given by $f_{0}=\frac{1}{2\pi }\sqrt{\frac{(k_{e}-k_{ess)}}{m}}$, will decrease as electrostatic spring constant $K_{ess}$ increases. The spring constant will increase as the cross voltage between the stator and rotor increases. 

Finally, for the proposed MEMS structure the spring constant is 9.22 N/m, variant capacitance per driving voltage is 5 aF/V, the resonance frequency at $V_{dc}$ = 0 V is 18,796 kHz, the tuning range of resonance frequency is 16,379.14–18,796 Hz, and the maximum tuning range is 128,590.5 ppm.

## 5. Circuit Design and Simulation

### 5.1. System Architecture

**Figure 9:**
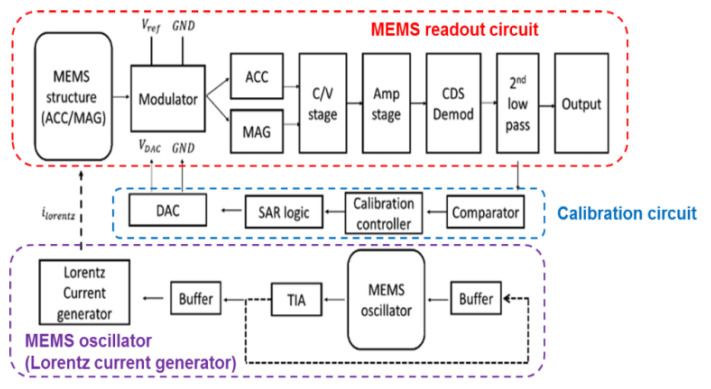
The overall architecture of the proposed readout system.

The system architecture, shown in Figure 9, is divided into MEMS readout circuit, calibration circuit, and Lorentz current generator (MEMS oscillator).

### 5.2. Readout Circuit

The readout architecture is shown in Figure 11 [2]. When the MEMS sensor is driven by the external acceleration or magnetic field, the rotor will move and cause varied capacitance. The variation of capacitance is modulated with a 400 kHz pulse signal and converted into a voltage signal by the capacitance-to-voltage (C/V) circuit, and the nested chopper amplifier is used to reduce the residual offset [23]. A fully differential bridge capacitive sensing scheme is used [11]. After the first demodulation, the signal band is converted to about 25 kHz and the second-order biquad filter separates the demodulated and undesired signals.

After the correlated double-sampling (CDS) with the demodulation function circuit, the amplitude of the signal will be larger by two times [11]. There should be a buffer to push the large capacitor [24], and two cascading RC filters are implemented for the filtering function. Then the frequency division multiplex reads multiple signals at once and modulates this signal to a different frequency. The time-division multiplexing (TDM) can reduce the mutual interference of the MEMS three-axis signals and the power consumption can be reduced.

### 5.3. Calibration Circuit

The settling time of calibration operation of node A in the two cascading RC filters is used to execute the calibration circuit. The calibration circuit eliminates the DC offset of the output resulting from process variation and includes the two cascading filters. Node A is the settling time of the calibration operation, the continuous-time comparator determines the polarity of the offset, the control logic circuit controls the switches to reduce the DC offset, the interface of calibration and readout circuits uses successive-approximate register (SAR) based logic to switch the unbalanced capacitance and reduce the DC deviation from differential input, and a 5-bit resistor-to-resistor (R-2R) digital-to-analog (DAC) process provides the analog output and connects to the control logic circuit as a unity gain voltage follower. After the first (coarse) operation, the second (fine) operation of the calibration circuit will complete 10 calibration cycles [2,25].

### 5.4. Resonator Circuit

**Figure 10:**
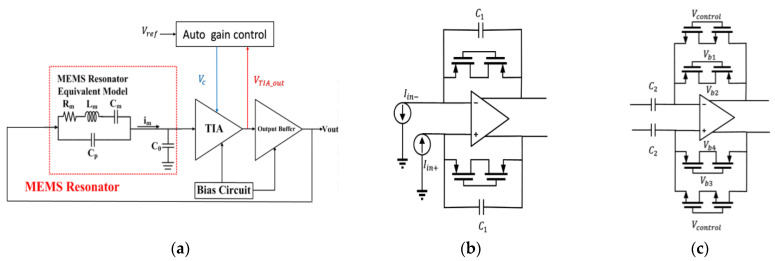
Architecture of (**a**) MEMS oscillator sustaining amplifier, (**b**) integrator, and (**c**) differentiator.

A MEMS resonator cascaded with a multi-stage trans-impedance amplifier (TIA) and output buffer to form a closed loop is shown in Figure 10a. To overcome the resistive loss (Rm) in the MEMS structure, a sustaining amplifier with high gain is required. The automatic gain control circuit is added to control the linearity of the output signal. To maintain the oscillation, the loop gain of the system must be higher than unity with zero phase shift to satisfy Barkhausen’s criterion.

The first stage of TIA is implemented by a topology of an integrated differentiated-based TIA and uses the advantage of high gain with sufficient bandwidth; the integrator and differentiator are shown in Figure 10b,c. The integrator first presents a phase shift of 90° and the differentiator compensates 90° of phase shift back. It results in a total phase shift approximately close to zero with high gain. The overall gain of the TIA is given by $A_{v}\left(s\right)≅\frac{-R_{1}}{1+sC_{1}R_{1}}*\left(-sC_{2}R_{2}\right)=\frac{sC_{2}R_{1}R_{2}}{1+sC_{1}R_{1}}$, where $R_{1},R_{2}$ represent the effective resistance of the MOS in the integrator and differentiator by varying the $V_{control}$ in Figure 10b, and the gain of TIA will be changed as $R_{2}$ is varied, with the $V_{control}$ of Figure 10c used to control the gain of the differentiator. The simulation result at a frequency of 18.8 kHz for the TT, SS, and FF corners is shown; the integrator gain is 165.27, 165.32, and 165.21 dB, the integrator phase is 89.76, 89.75, and 89.79°, the TIA gain is 145.2, 145.68, and 144.35 dB, and the TIA phase is −0.65, −0.65, and −0.61°.

**Figure 11:**
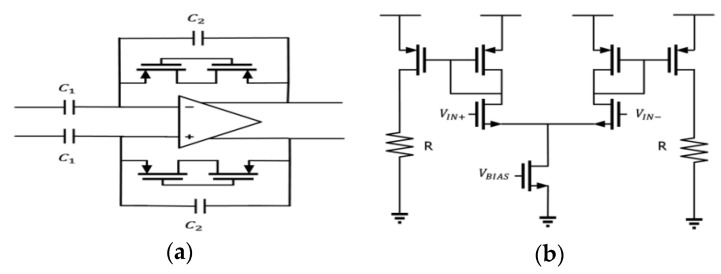
Circuit of (**a**) amplifying stage and (**b**) output stage.

Since the gain of the sustaining amplifier is still far behind the order of giga-ohms, a capacitor feedback amplifier (Figure 11a) is used to amplify the signal. In addition, a current-mirror amplifier with resistive load is used as the output stage (Figure 11b). With this, the output swing of the amplifier can be wide from 0.2 to 1.6 V. The simulation results are shown at a frequency of 18.8 kHz for the TT, SS, and FF corners; the sustaining amplifier gain is 197.72, 198.41, and 196.67 dB, the sustaining amplifier phase is −1.18, −1.19, and −1.1°, and the output DC level of the sustaining amplifier is 1.07, 1.13, and 1.01. Since there is no feedback loop in the output stage to lock the output DC level, the level will deviate. To solve the problem, a decoupled capacitor is used to block the DC part and pass the AC part of the signal.

**Figure 12:**
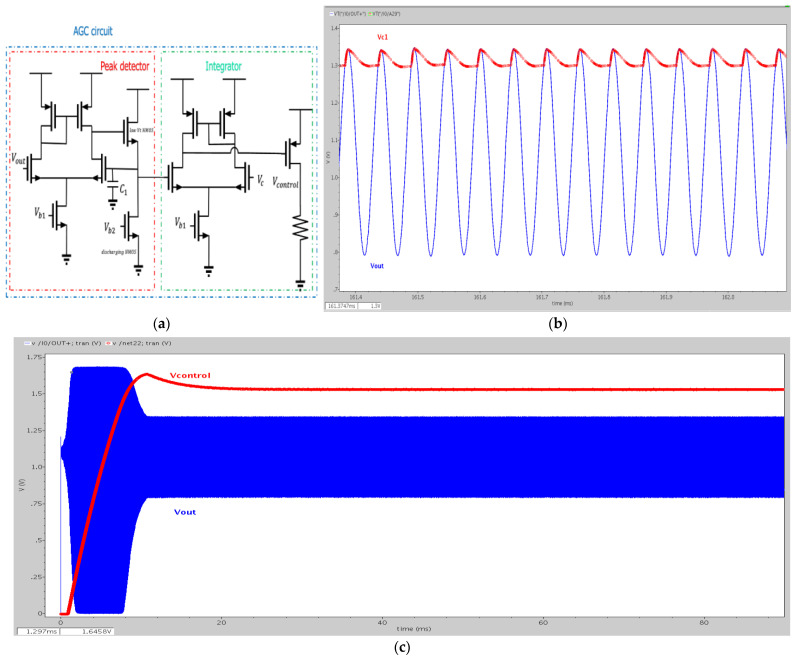
(**a**) The automatic gain control circuit, (**b**) after peak detector, (**c**) after integrator.

An automatic gain control (AGC) circuit is used to control the output swing of the MEMS oscillator by giving a reference voltage. The AGC circuit consists of a peak detector and an integrator. The operating principle is illustrated in Figure 12a. $V_{out}$; the output voltage of the oscillator is comparable to the voltage stored on $C_{1}$ with an amplifier. If $V_{out}$ is greater than $V_{C1}$, the output of the amplifier will be raised and close to the supply rail ($V_{DD}$). The voltage on $C_{1}$ will increase because of the charging current from the low-threshold voltage n-type MOS (NMOS) device. Besides, if $V_{out}$ is smaller than $V_{C1}$, $V_{C1}$ will be pulled down to the ground by the discharging NMOS. Thus, by balancing the charging and discharging speedwell, $V_{C1}$ can follow the peak value of $V_{out}$.

Similarly, in the integrator, we compare the voltage on $C_{1}$ and the reference voltage $V_{c}$. If $V_{c1}$ is greater than $V_{c}$, it means the amplitude of the oscillator is higher than the expected value, and the output of the integrator will be pulled down and $V_{control}$ will raise. As shown in Figure 10c, the gain of the differentiator can be changed since $V_{control}$ is raised. Because the conductance resistance is reduced when the gate voltage of NMOS increases, the gain of the TIA will decrease and lower the amplitude of the MEMS oscillator. Finally, the peak value of $V_{out}$ will be close to the reference voltage $V_{c}$. Figure 12b,c shows simulation results of the AGC circuit, and the reference voltage is 1.3 V.

## 6. Measurement and Discussion

**Figure 13:**
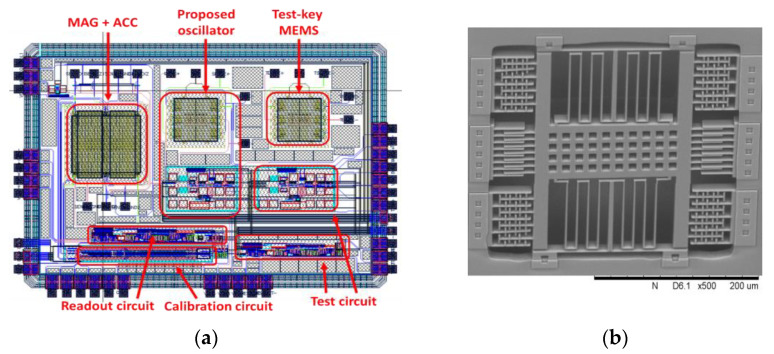
(**a**) Layout view of chip; (**b**) scanning electron microscope view of proposed MEMS oscillator.

Figure 13a shows a layout view of the chip. The ACC and MAG are combined in one structure. The MEMS structure in the middle is the proposed MEMS oscillator. The testing circuits of the readout circuit and sustaining amplifier are included in the chip. The dimensions of the chip are 2538.64 × 1849.37 µm^2^. Figure 13b shows the *scanning* *electron* *microscope* (SEM) view of the proposed MEMS oscillator.

**Figure 14:**
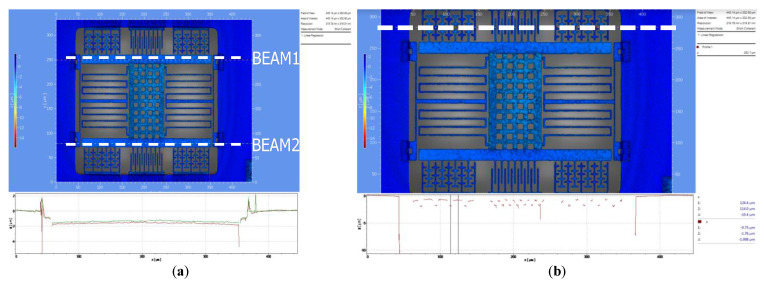
White light interferometry (WLI) view of (**a**) structure along beams on two sides and (**b**) structure along stator and rotor.

Figure 14a shows the top view of the MEMS oscillator captured by white light interferometry (WLI), and the displacement along the z-axis between the two beams on the sides is only 0.1 µm. As shown in Figure 14b, the curvature at the anchor is 0.351 µm. The distance between stator and rotor, which determines the overlapping area of electrodes, is only 1.008 µm. The measurement result shows that the fishbone finger will maintain the flatness of the structure. The maximum displacement caused by curvature along the z-axis is 2.385 µm in the middle of the finger, 0.618 µm on the sides of the finger, 1.5 µm in the springs, and 1.775 µm in the beam.

The frequency responses of the MAG simulation, measurement, and error percentage are 17.62 kHz, 17.99 kHz, and 2.06% for in-plane resonance frequency, 18.87 kHz, 18.58 kHz, and 1.56% for out-plane resonance frequency, 25.33 kHz, 395.8 kHz, and 93.6% for in-plane *Q* value, and 155.57 kHz, 142.29 kHz, and 9.3% for out-plane *Q* value. The frequency responses of the MEMS oscillator simulation, measurement, and error percentage are 18.8 kHz, 19.5 kHz, and 3.49% for in-plane resonance frequency and 183 kHz, 134.8 kHz, and 36% for in-plane *Q* value.

**Figure 15:**
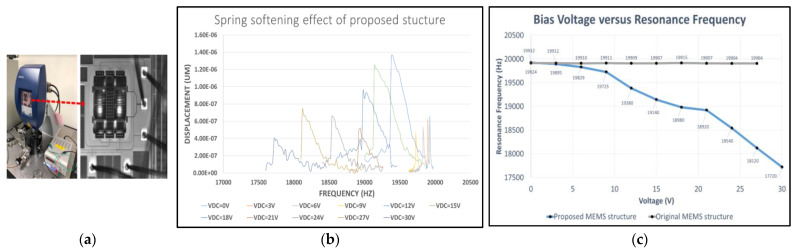
(**a**) Measurement setup of spring softening effect; (**b**) spring softening effect of proposed structure; (**c**) com-parison of MEMS oscillator with fishbone and original.

The measurement setup of the spring softening effect is shown in Figure 15a. The measurement result of the proposed MEMS oscillator with bias voltage changed from 0 to 27 V, where the resonance frequency is reduced by only 2204 Hz. The frequency tuning ranges of the proposed oscillator for simulation and measurement are 16,379.14–18,796.1 and 17,720–19,924 Hz, and the tuning ranges for simulation and measurement are 128,590.5 and 110,620.36 ppm. 

**Figure 16:**
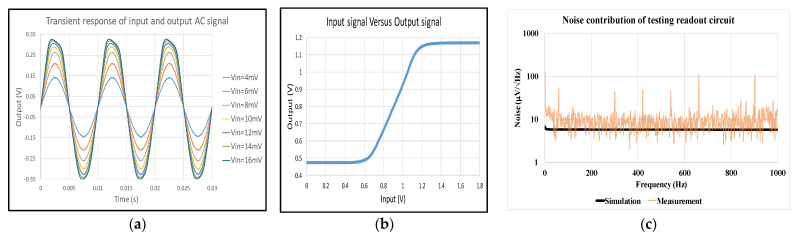
(**a**) Output waveforms corresponding to increasing input at 100 Hz; (**b**) input versus output signal of readout circuit; (**c**) noise contribution of testing readout circuit.

The measurement of the circuit is on the printed circuit board (PCB), and the low dropout regulator (LDO) and TIA are used to connect with the MEMS oscillator and amplify the small output current sensed from the electrodes. Then, the testing circuit of the readout circuit is measured. To model capacitance variation with 100 Hz caused by acceleration and 18,800 Hz caused by Lorentz current, 100 and 18,800 Hz sinusoidal test signals with a magnitude of 4 mVpp are given. Figure 16a,b shows the output waveforms and output range corresponding to varied input. The output range is from about 0.5 to 1.13 V, which is not an overestimation compared to the measurement.

The contribution of noise is shown in Figure 16c. The noise floor of the testing readout circuit is equal to 11.967 $\mu V/\sqrt{\mathrm{Hz}}$ compared to 5.72 $\mu V/\sqrt{\mathrm{Hz}}$ in the simulation. Comparing the gain in DC and periodic AC analysis (PAC), the simulation, measurement, and error results are 36.1 dB, 41.76 dB, and 13.55%; the 3 dB bandwidths are 54 kHz, 53.34 kHz, and 1.23%; the output upper bounds are 1.2 V, 1.13 V, and 6.37%; the output lower bounds are 0.48 V, 0.5 V, and 3.68%; and the noise floor values are 5.72 $\mu V\sqrt{\mathrm{Hz}}$, 11.97 $\mu V/\sqrt{\mathrm{Hz}}$, and 52.2%.

**Figure 17:**
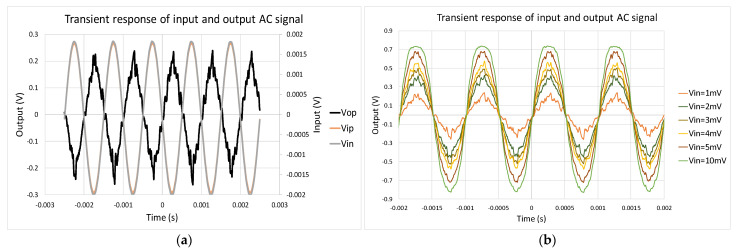
(**a**) Input and output signals under 1 kHz, 0.1 mVpp difference sinusoidal input; (**b**) output waveforms corresponding to increasing input at 1 kHz.

For the TIA test circuit of the sustaining amplifier in the MEMS oscillator, there should be an input current for measuring the gain of the TIA. Hence, an on-chip Gm-cell is implemented to provide a small input current. The simulated trans-conductance of the Gm-cell is about 15 A/V. As a result, by inputting voltage with a differential of 0.1 mV, an input current equal to 1.5 nA can be obtained. We gave differential inputs of 1 kHz sinusoidal wave with 0.1 mV difference as the testing signal. Figure 17a shows the measurement of the output signal under the testing input. Figure 17b shows the output voltage under different inputs. Because of the high gain, the noise in the input will be amplified and disturb the output. To calculate the gain of the TIA, root mean square is used, and the gain can be evaluated by $Gain_{TIA}=\left|\frac{Vout_{rms}}{Vin_{rms}}\right|$. The gain of the tested TIA circuit for simulation, measurement, and error is 122.14 dB, 128.903 dB, and 5.24%.

Comparisons of the MEMS magnetometer are shown in Table 4, the sensitivity and resolution for the previous paper [2] in the three axes were 7.1–10.7 uV/uT and 44.06–87.46 nT/√Hz. The sensitivity and resolution in this paper were 7.5–218.3 uV/uT and 3.032–96 nT/√Hz. References [4,5,6,7] proposed to use different structures and different from the CMOS MEMS process.

Comparisons of the MEMS oscillator (OSCI) are shown in Table 5, defining a figure of merit (FOM) [10]. The resonance frequency of the MEMS oscillator (OSCI) at *V_dc_* = 0 V is 18,796 kHz, and the tuning range of resonance frequency is 16,379.14–18,796 Hz. The maximum tuning range is 128,590.5 ppm, better than other papers.

## 7. Conclusions

The study integrates MAG and ACC in one multi-function MEMS structure, then uses a MEMS oscillator to enhance the sensitivity and resolution of the three-axis MAG. The sensitivities in the three axes improve from 7.1–10.7 uV/uT to 7.5–218.3 uV/uT and the resolutions from 44.06–87.46 nT/√Hz to 3.032–96 nT/√Hz.

The MEMS oscillator with bias voltage changed from 0 to 27 V, and the resonance frequency was reduced by only 2204 Hz, less than the ring oscillator.

In the MEMS sensor, three solutions for curvature are presented. One is the proposed MEMS structure with fishbone, and the curvature along the z-axis is improved from 7.5 to 2.38 µm and 1.8 to 0.618 µm for sensing electrodes in the middle and sides. The second solution is the readout circuit using noise-reduction technology, the frequency division multiplexing method, and the time-division multiplexing method. The third solution is implementing a capacitance calibration circuit. A SAR-based calibrator can eliminate the offset between differential outputs with a minimum capacitive resolution of 20.83 aF, and the offset caused by the curvature can be greatly reduced from hundreds of millivolts down to below 10 mV after the calibration.

In addition, the multi-function MEMS with oscillator can be placed at an ambient pressure of 10 torr, which can enhance the *Q* value by 10 times, increase the displacement, and lower the damping coefficient by one-tenth. The sensor can have a larger vibration amplitude when there is magnetic field strength and can obtain a larger capacitance change value, which can also effectively reduce the sensing size to produce less residual stress.

## Figures and Tables

**Table 1:** Sizes of MEMS magnetometer.

Position	Value	Position	Value
Proof mass length (μm)	278.2	Stator fingers (D) (μm)	62.4
Proof mass width (μm)	23.4	Rotor fingers (E) (μm)	62.4
Finger width (A) (μm)	2.6	Spring width (F) (μm)	2.6
Finger gap width (B) (μm)	2.6	Spring width parallel (G) (μm)	2.6
Finger overlap length (C) (μm)	59.8	Spring width perpendicular (H) (μm)	7.8
Finger overlap length (μm)	-	Spring length(I) (μm)	140.4

**Table 2:** Sizes of proposed differential MEMS oscillator.

Position	Value	Position	Value
Area (μm^2^)	348 × 346.13	Length of rotor fingers (μm)	37.5, 42.5
Length of shutter (μm)	294.53	Width of springs (μm)	2.5
Width of shutter (μm)	188	Length of springs (μm)	104.1
Finger gap spacing (μm)	2.5, 5	Gap spacing between springs (μm)	10.4
Finger overlap length (μm)	25, 37.5	Turns of springs (μm)	2 × 4
Length of stator fingers (μm)	37.5, 47.5	Number of driving combs	14 × 2
		Number of sensing combs	16 × 4

**Table 3:** Simulation result of capacitance array. Sense+, positive sensing port; Sense−, negative sensing port; Drive+, positive driving port; Drive−, negative driving port.

Initial Capacitance (fF)	Shuttle	Sense+	Sense−	Drive+	Drive−
Shuttle	-	45.374	45.374	21.555	21.555
Sense+	45.374	-	0.729	1.156	1.157
Sense−	45.374	0.729	-	1.157	1.156
Drive+	21.555	1.156	1.157	-	0.073
Drive−	21.555	1.157	1.156	0.073	-

**Table 4:** Comparison of MEMS magnetometer.

Specification	[4]	[13]	[5]	[6]	[7]	[2]	This Paper	This Paper	This Paper
Type	MAG	MAG	MAG	MAG	MAG	MAG	MAG	MAG	MAG
Axis	x/y/z	x/y/z	y	Three	z	Three	x	y	z
Sensitivity (μV/μT)	0.13/0.14/1.51	-	2.1 × 10^5^	250	150	7.1 ~ 10.7	218.3	74.33	7.5
Resolution (nT/√Hz)	319.9/296.5/121.6	344/344/285	0.00276	4	2080	44.06 ~ 87.46	3.302	9.69	96
Mag drive current (mA)	4.18/4.02/4.02	4.5	7.245	1.08	0.25	2	2	2	2

**Table 5:** Comparison of MEMS oscillator (OSCI). CMOS: complementary metal-oxide semiconductor.

Specification	[3]	[8]	[9]	[10]	This Paper
Type	OSCI	OSCI	OSCI	OSCI	OSCI
Technology	Silicon on Insulator(SOI)	0.35 μm CMOS-MEMS	0.35 μm CMOS-MEMS	0.35 μm CMOS-MEMS	0.18 μm CMOS-MEMS
Resonance frequency (MHz)	0.327	0.327	1.23	3.2	0.188
FOM ^a^ (Hz^2^Ω^2^) (× 10^20^)	-	-	2.04	3.16	4.75
Frequency tuning range (ppm)	6500	50,000	-	-	110,620

^a^ FOM: Figure of merit.

## Data Availability

Relevant information can be obtained through the laboratory.
